# An Activity‐Reinforced P‐Modified Single‐Atom Cu Nanozyme Potentiates IDO Blockade‐Induced Tumor Radiotherapy

**DOI:** 10.1002/advs.202524253

**Published:** 2026-10-31

**Authors:** Wei Zhao, Songliu Hu, Zhanggui Wang, Liqun Wang, Liming Zhang, Qi Li, Hua Tang, Yang Zhong, Zhongxian Zhang, Kelong Fan, Shipeng Ning, Xiyun Yan

**Affiliations:** ^1^ Research Center of Nanomedicine Technology The Second Affiliated Hospital of Guangxi Medical University Nanning China; ^2^ Harbin Medical University Cancer Hospital Harbin China; ^3^ Department of Radiotherapy Cancer Center Aerospace Center Hospital Beijing China; ^4^ Department of Radiation Oncology Anhui No. 2 Provincial People's Hospital Hefei China; ^5^ State Key Laboratory of Metabolic Dysregulation & Prevention and Treatment of Esophageal Cancer Tianjian Laboratory of Advanced Biomedical Sciences Academy of Medical Sciences Zhengzhou University Zhengzhou China; ^6^ CAS Engineering Laboratory For Nanozyme State Key Laboratory of Biomacromolecules Institute of Biophysics Chinese Academy of Sciences Beijing China

**Keywords:** 3‐dioxygenase, breast cancer, indoleamine 2, phosphorus modification, radiotherapy, single‐atom nanozyme

## Abstract

Radiotherapy exerts both immunostimulatory and immunosuppressive effects on breast cancer (BRCA), limiting its therapeutic potential. To shift this balance toward a more favorable antitumor immune response, an indoleamine 2,3‐dioxygenase 1 (IDO1)‐blocking nanozyme specifically designed to potentiate tumor radioimmunotherapy was developed. Using a ZnO–carbon nanoframework, single Cu–N_4_ catalytic sites were engineered to construct a single‐atom Cu nanozyme (SAzyme) exhibiting dual peroxidase‐ and catalase‐mimicking activities for the delivery of the IDO1 inhibitor 1‐methyl‐tryptophan (1‐MT), resulting in a platform termed CPMR. Phosphorus modification further improved the catalytic performance of the SAzyme. Once internalized by tumor cells, CPMR generates sufficient oxygen to alleviate hypoxia and improve radiosensitivity while simultaneously inducing oxidative stress to promote tumor cell death and trigger immunogenic cell death. These events facilitate the recruitment and activation of tumor‐infiltrating cytotoxic T lymphocytes. The controlled release of 1‐MT inhibits IDO1 activity and reduces L‐tryptophan levels, suppressing regulatory T‐cell function and mitigating the immunosuppressive tumor microenvironment. These mechanisms synergistically improve the efficacy of radiotherapy against BRCA. CPMR represents a promising tumor‐reprogramming strategy capable of eliciting a robust antitumor immune response and significantly strengthening radiotherapeutic outcomes in patients with breast cancer.

## Graphical abstract

A phosphorus‐modified single‐atom copper nanozyme delivering the IDO1 inhibitor 1‐methyl‐tryptophan enhances breast cancer radioimmunotherapy by simultaneously relieving hypoxia and inducing immunogenic cell death through dual enzyme activities while blocking tryptophan metabolism to suppress regulatory T cell function.

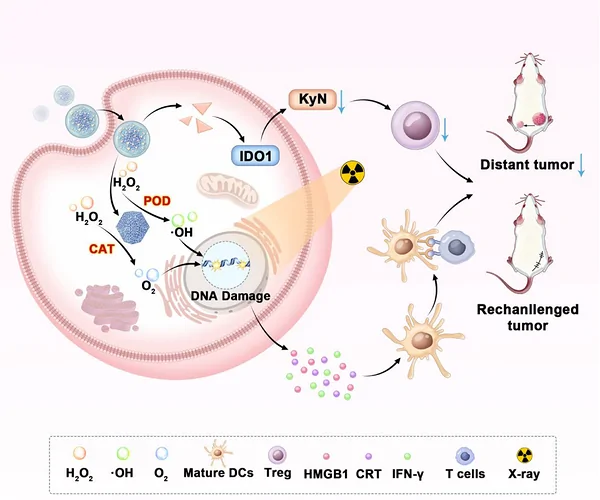

## 1 Introduction

Radiotherapy (RT), an indispensable component of multimodality treatment for breast cancer (BRCA), exerts dual and often opposing influences on the tumor immune microenvironment [1, 2, 3, 4, 5]. On the one hand, ionizing irradiation eliminates residual tumor cells through the induction of DNA double‐strand breaks. Concurrently, RT initiates immunogenic cell death (ICD), enabling antigen presentation and activation of T lymphocytes to strengthen antitumor immunity [6]. On the other hand, RT induces elevated levels of immunosuppressive cytokines and chemokines that recruit large numbers of myeloid inflammatory cells, fostering revascularization and DNA repair within residual tumor tissues. RT has also been shown to activate forkhead box protein 3 (FOXP3)‐expressing regulatory T (Treg) cells, whose immunosuppressive activity is closely associated with poor patient prognosis [7]. Moreover, the immunosuppressive tumor microenvironment (TME) often limits the therapeutic benefit of RT, necessitating higher radiation doses for tumor control, an approach constrained by the risk of damage to adjacent normal tissues [8, 9].

Indoleamine 2,3‐dioxygenase 1 (IDO1) activity is increased in tumor cells and blood samples from BRCA patients [10]. Its upregulation is driven by proinflammatory cytokines, and within the TME, IDO1 is expressed predominantly in tumor cells, dendritic cells (DC), and macrophages. IDO1 plays a critical role in BRCA progression by catalyzing the conversion of L‐tryptophan (Trp) to L‐kynurenine (Kyn), simultaneously depleting Trp and increasing the level of intracellular Kyn [11]. Trp exhaustion compromises lymphocyte function, diminishing their capacity to attack tumor cells. A reduced Trp/Kyn ratio further impairs effector T‐cell activity and increases immunosuppressive FOXP3 expression in CD4^+^ T cells [12]. Through these mechanisms, IDO1 drives profound cancer‐associated immunosuppression and facilitates tumor metastasis [13]. Recently, IDO1 inhibitors have been integrated with other therapeutic modalities to reverse the immunosuppressive TME and restore antitumor immunity [14, 15, 16]. Among them, 1‐methyltryptophan (1‐MT), a competitive IDO‐specific inhibitor, has demonstrated strong potential in modulating tumor‐induced immunosuppression [17, 18]. Combining IDO1 inhibition with RT has shown promise in depleting Tregs; however, the inherently low immunogenicity of certain BRCA tumors continues to restrict the effectiveness of such combination strategies.

Low immune‐cell infiltration in the TME reduces tumor immunogenicity and weakens host immune responses [19]. When tumor cells undergo ICD, they acquire antitumor immunogenicity, transforming immunosuppressive “cold” tumors into immunogenic “hot” tumors and increasing RT sensitivity [3, 8]. Owing to the strategic design of nanomaterial systems, noninvasive nanozymes have attracted significant interest for their capacity to induce ICD through tailored catalytic mechanisms [20, 21, 22, 23, 24]. Single‐atom nanozymes (SAzymes) offer exceptional catalytic performance because of the optimized modification environment surrounding isolated metal sites [25, 26, 27, 28, 29]. SAzymes based on metals such as copper, gold, and platinum have been reported to display robust enzyme‐mimicking activities [30, 31]. By leveraging elevated intracellular H_2_O_2_ in tumors, catalase (CAT)‐mimicking nanozymes can alleviate hypoxia and improve tumor responsiveness to therapy. Peroxidase (POD)‐mimicking nanozymes convert H_2_O_2_ into cytotoxic reactive oxygen species, disrupting redox homeostasis, amplifying oxidative stress, and driving ICD [32]. This process facilitates the release of multiple damage‐associated molecular patterns (DAMP), including high‐mobility group box 1 (HMGB1) and calreticulin (CRT), which increase antigen presentation via DC maturation and activate both innate and adaptive immune responses. These mechanisms increase tumor sensitivity to treatment and strengthen systemic antitumor immunity. However, catalytic activity can be limited by insufficient SAzyme density [33].

Despite advances in radiotherapy and immunotherapy, BRCA tumors often exhibit low immunogenicity and an immunosuppressive microenvironment, limiting therapeutic efficacy [34]. To address this challenge, a multifunctional Cu‐based single‐atom nanozyme (CPMR) was developed that combines dual CAT‐ and POD‐like catalytic activity with IDO1 inhibition, simultaneously increasing immunogenic cell death, reversing immunosuppression, and improving radiosensitization. This integrated strategy aims to overcome tumor resistance, amplify antitumor immunity, and reduce recurrence and metastasis in breast cancer models. Initially, a Cu SAzyme containing single Cu‐N_4_ sites within a ZnO–carbon nanoframework was synthesized (Scheme 1). Subsequent decoration with phytic acid, followed by annealing, yielded a nanozyme termed CP. Phosphorus modification around single Cu atoms significantly increased the catalytic activity of the SAzyme. The IDO1 inhibitor 1‐methyl‐tryptophan (1‐MT) was then loaded onto the porous Cu SAzyme through physical adsorption. To improve tumor‐specific targeting, an arginine‐glycine‐aspartic acid (RGD) ligand was conjugated onto the nanozyme surface, producing the final construct, termed CPMR.

**SCHEME 1:**
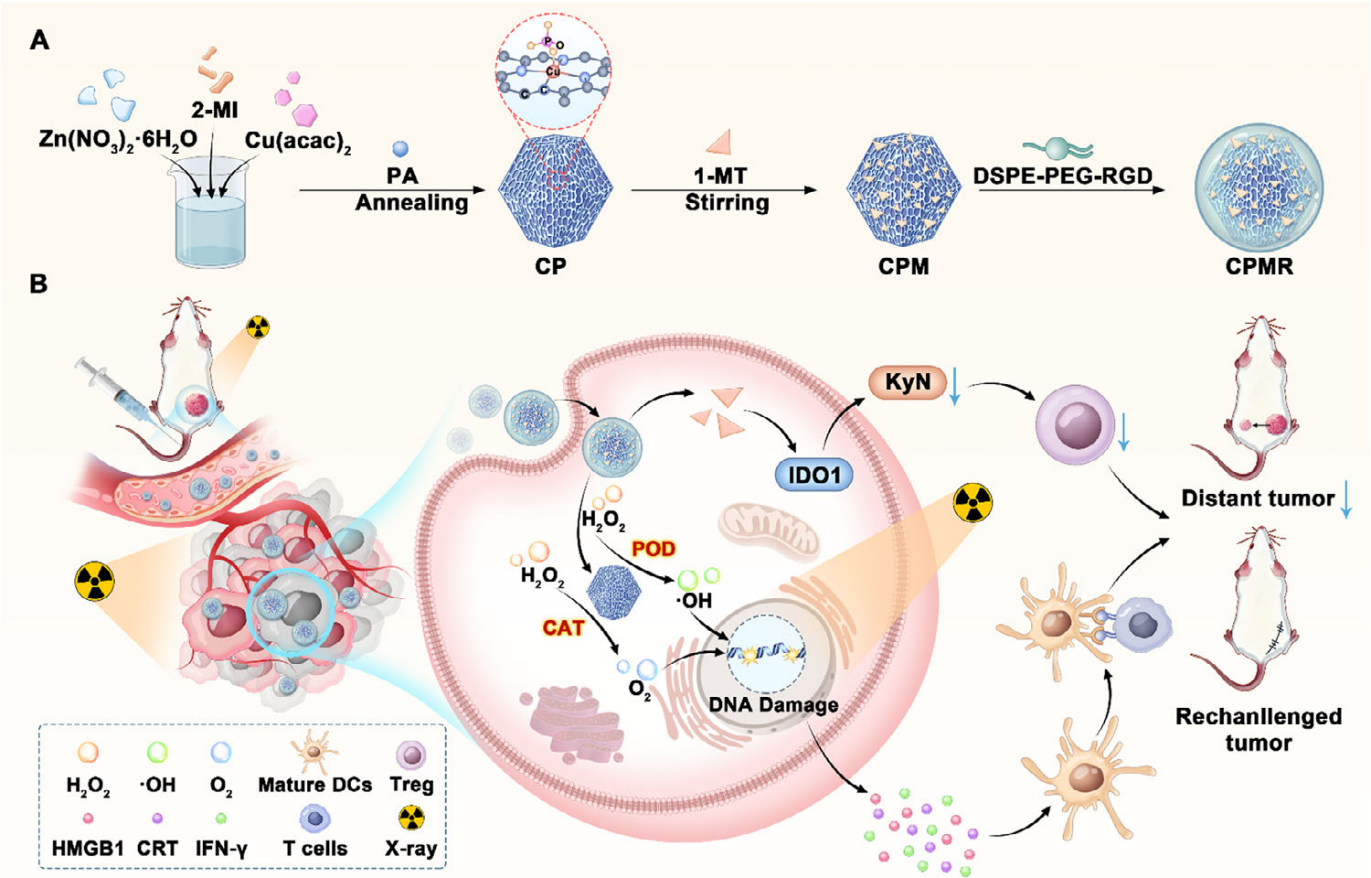
Schematic illustration of CPMR synthesis and its radiosensitization mechanism. (A) Stepwise synthesis of the CPMR nanozyme, including single‐atom Cu incorporation, phosphorus modification, 1‐MT loading, and RGD functionalization. (B) Mechanistic overview of CPMR‐mediated modulation of the tumor immune microenvironment, promotion of immunogenic cell death, increased T‐cell responses, and improved radiotherapy efficacy to inhibit breast cancer recurrence and metastasis.

Following intravenous administration, CPMR selectively accumulated in tumor tissues, where it generated abundant oxygen from endogenous H_2_O_2_ within the TME, relieving hypoxia and improving RT‐induced radiosensitization. The POD‐like catalytic activity of CPMR induced a surge in oxidative stress, generating cytotoxic ·OH radicals that promoted ICD, facilitated DC maturation, and activated T‐cell responses to improve tumor immunotherapy. Similarly, 1‐MT released from the CPMR effectively suppressed Treg activity by inhibiting IDO1, contributing to the reversal of immunosuppression within the TME. These integrated mechanisms increase the sensitivity of BRCA tumors to ionizing irradiation and significantly reduce tumor recurrence and metastasis.

## 2 Results and Discussion

### 2.1 Synthesis and Characterization of CPMR

First, the CPMR nanozyme was synthesized using a Cu‐N_4_ structure, which is consistent with previously reported strategies. Representative transmission electron microscopy (TEM) images of CP and CPR were shown in Figures S1 and S2. The TEM image (Figure 1A) and corresponding diameter distribution (Figure 1B) show that the CPMR possesses a uniform polyhedral morphology with an average diameter. Elemetal mapping verified the well‐distribution of Cu, C, N, P and O in CPMR (Figure 1C). To assess the colloidal stability of the CPMR, the zeta potential was monitored over seven days. No evident change was observed, indicating that CP, CPR and CPMR remained stable (Figure 1D). The infrared characteristics of CMPR, as shown in the Fourier transform infrared (FTIR) spectra in Figure 1E, were essentially identical to those of DSPE‐PEG‐RGD. This occurred because DSPE‐PEG‐RGD completely encapsulated CPM and tryptophan, forming a core‐shell‐like structure. However, a noticeable increase in the relative intensity of the O‐H and N‐H absorption peaks at 3438 cm^−^^1^ was observed, along with shifts and broadening of the absorption peaks at 1252 cm^−^^1^ and 1110 cm^−^^1^. These spectral changes indicated the formation of a new hydrogen‐bonding network between 1‐MT and DSPE‐PEG‐RGD during the polymerization process. FTIR spectra in Figure S3 confirmed that the characteristic peaks of 1‐MT remained unchanged after exposure to the acidic microenvironment in existence of H_2_O_2_ mimicking TME. This new network altered the electronic environment around the polar groups, leading to shifts and broadening of the relevant absorption peaks. These subtle changes served as indirect evidence for the successful incorporation of 1‐MT into CMPR and its participation in the formation of the composite system with DSPE‐PEG‐RGD. In addition, UV–vis absorption spectra further verified the successful loading of 1‐MT onto CPMR (Figure 1F). The releasing behavior of 1‐MT from CPMR in TME mimicking solution was investigated where rapid releasing profile was shown in Figure S4. Atomic force microscopy (AFM) measurement was performed to examine the interfacial microstructure as shown in Figure 1G, which demonstrating an average thickness of 27 nm. The elemental composition and valence states within CPMR were subsequently examined using x‐ray photoelectron spectroscopy (XPS). High‐resolution analysis of the P 2p spectrum revealed one distinct peak at 134.3 eV (Figure 1H). The N 1s spectrum (Figure 1I) exhibited characteristic signals corresponding to pyridinie‐N (397.7 eV), Cu–N (398.9 eV), pyrrolic‐N (399.9 eV), graphitic‐N (401.1 eV), and oxidized‐N (402.7 eV), demonstrating that N species effectively anchor Cu atoms *via* covalent bonding.

**FIGURE 1:**
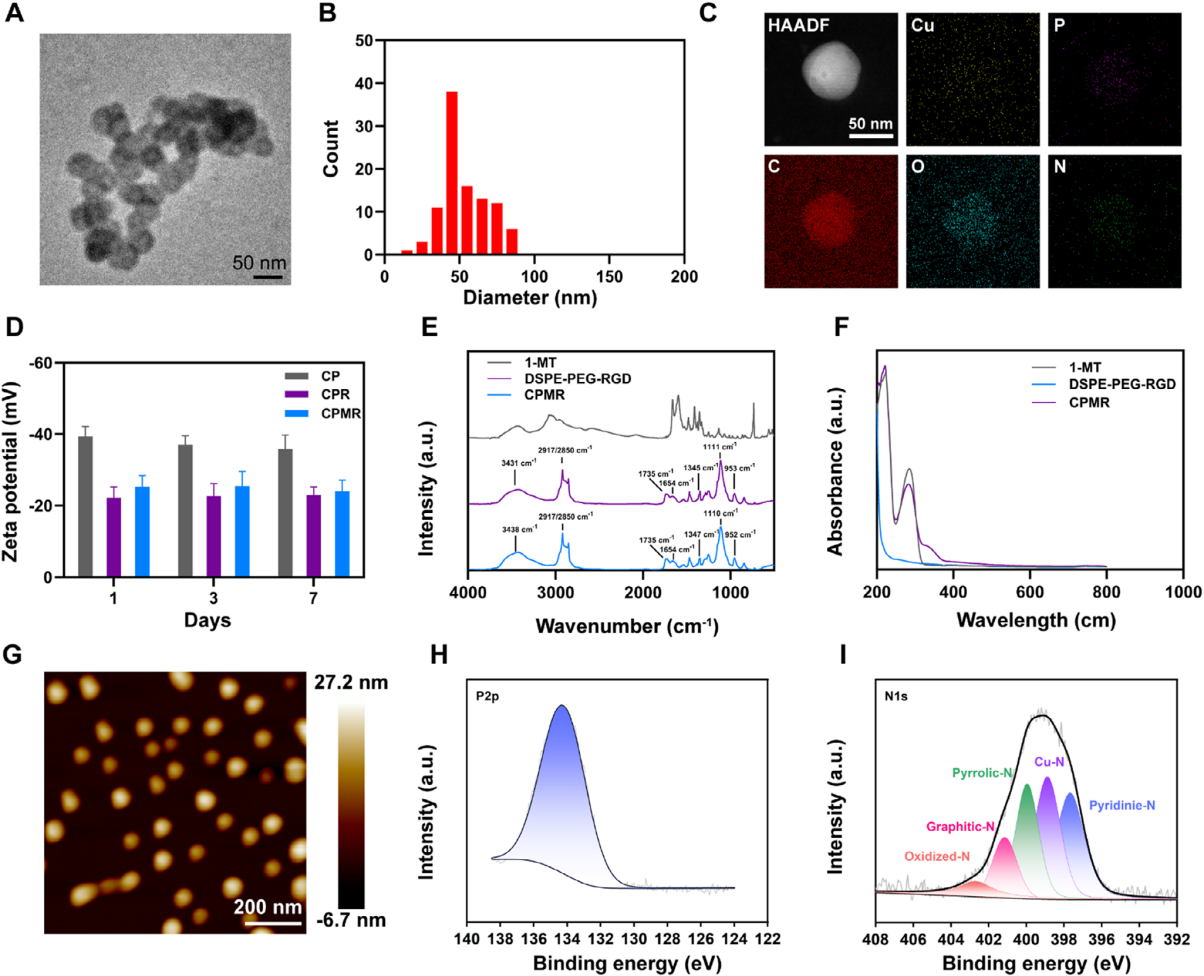
Structural and compositional characterization of the CPMR. (A) TEM image of the CPMR. (B) Particle diameter distribution. (C) HAADF‐STEM and corresponding elemental mapping (Cu, P, C, O, N) of CPMR. (D) Time‐dependent zeta potential of CP, CPR, and CPMR within 7 days (n = 3). (E) FTIR spectra. (F) UV‐vis absorptionspectralof1‐MT, DSPE‐PEG‐RGD and CPMR. (G) AFM analysis of CPMR. High‐resolution spectra of (H) P 2p and (I) N 1s for CPMR.

Next, the structural configuration of Cu‐N_4_ was analyzed via synchrotron‐radiation‐based x‐ray absorption fine structure (XAFS) spectroscopy. The normalized x‐ray absorption near‐edge structure (XANES) spectrum at the Cu K‐edge (Figure 2A) indicated that Cu was present in a high oxidation state. Fourier transformed extended x‐ray absorption fine structure (FT‐EXAFS) fitting of the Cu K‐edge (Figure 2B) revealed a modification environment consistent with a Cu‐to‐N ratio of approximately 1:4. In contrast to Cu foil, which exhibited a Cu–Cu peak at 2.23 Å, CP displayed a predominant Cu–N peak at 1.41 Å, confirming the formation of Cu–N_4_ modification (Figure 2C). Wavelet transform (WT) contour plots further distinguished the Cu–N modification in CP from the Cu–Cu scattering observed in the Cu foil (Figure 2D).

**FIGURE 2:**
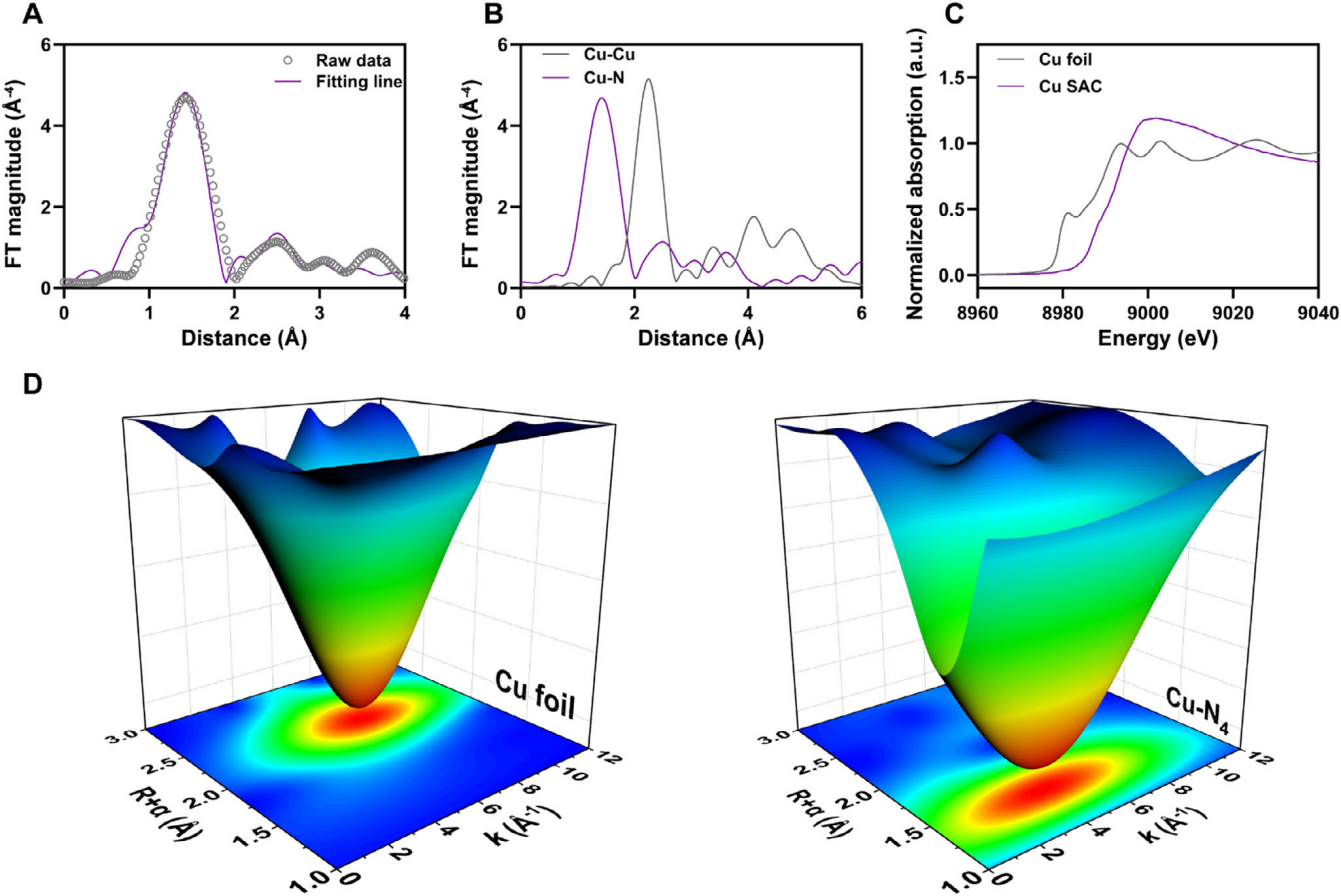
Atomic structural characterization of Cu sites in CPMR. (A) FT‐EXAFS spectrum at the Cu K‐edge, with raw data and fitting curve. (B) Simulated EXAFS signals of Cu‐Cu and Cu‐N scattering paths. (C) Cu K‐edge XANES spectra of Cu foil and Cu SAC. (D) Wavelet transform contour plots of the Cu foil and Cu SAC (Cu‐N_4_).

### 2.2 Enzyme Property Evaluation

Although SAzymes exhibit the advantage of high atomic utilization, their catalytic activity remains constrained by the relatively low density of active single‐atom sites. To improve the catalytic performance, phosphorus modification onto the Cu centers was introduced through an etching process. The CAT‐like activity of CP and Cu‐N_4_ was first evaluated by monitoring oxygen generation in the presence of H_2_O_2_. As shown in Figure 3A, Cu‐N_4_ produced a moderate amount of oxygen, reaching 2.3 mg/L O_2_ at 10 min. CP generated substantially higher oxygen levels, up to 5.2 mg/L at 10 min, demonstrating that phosphorus modification significantly improved the CAT‐mimicking capability of the SAzyme.

**FIGURE 3:**
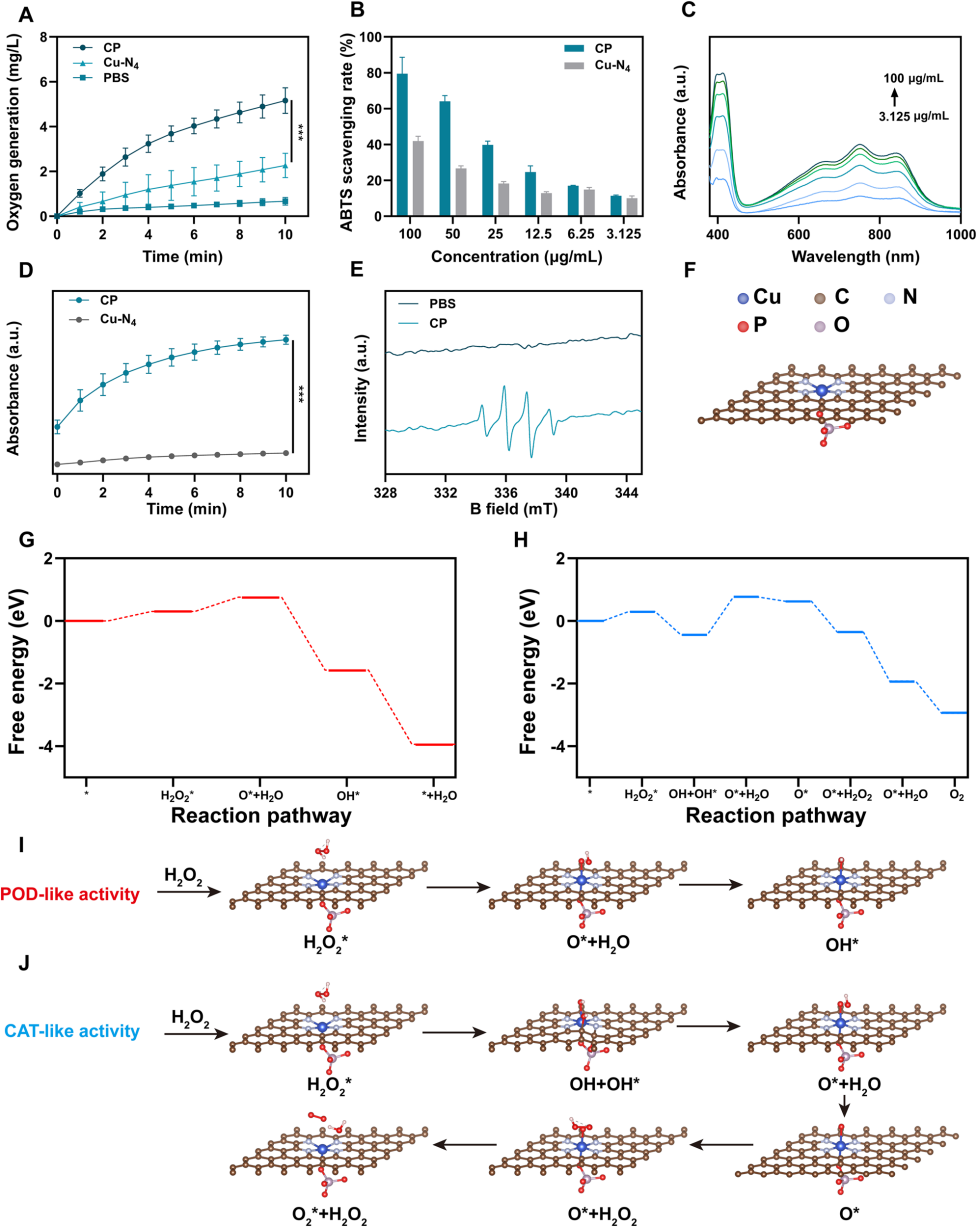
Enzymatic activity evaluation and DFT theoretical calculations. (A) Time‐dependent oxygen generation curves of CP, Cu‐N_4_ and PBS (n = 3). (B) Concentration‐dependent ABTS radical scavenging rates of CP and Cu‐N_4_ (n = 3). (C) UV–vis absorbance spectra of CP in the presence of TMB after reaction with H_2_O_2_. (D) Time‐dependent absorbance of TMB after reaction with CP and Cu‐N_4_ in the presence of H_2_O_2_ (n = 3). (E) ESR spectroscopy detection of •OH radical generation. (F) Schematic illustration of Cu‐N_4_ surface modification. (G) Reaction mechanism and relative Gibbs free energy changes for POD‐like activity based on DFT calculations. (H) Reaction mechanism and relative Gibbs free energy changes for CAT‐like activity based on DFT calculations. Schematic diagram of (I) POD‐like activity and (J) CAT‐like activity. Statistical significance was determined by one‐way ANOVA with a Tukey post hoc test. ^***^ *P* < 0.001.

To assess the free radical scavenging ability of nitrogen, 2,2′‐azino‐bis(3‐ethylbenzothiazoline‐6‐sulfonic acid) (ABTS) was employed as the chromogenic substrate to quantify the scavenging capacity of CP and Cu‐N_4_ at various concentrations. As illustrated in Figure 3B, both materials exhibited concentration‐dependent ABTS scavenging ability. However, compared with Cu‐N_4_, CP exhibits a lower ABTS scavenging capacity, indicating weaker antioxidant capacity and thus implying that CP has stronger POD‐like activity. A complementary chromogenic assay using 3,3′,5,5′‐tetramethylbenzidine (TMB) further confirmed the concentration‐dependent POD‐like reactivity of CP (Figure 3B). Consistent with the ABTS results, ·OH generation increased steadily within 10 min and was strongly positively correlated with POD‐like catalytic activity (Figure 3D). Electron spin resonance (ESR) analysis further confirmed that ·OH production takes place more efficiently under acidic conditions. These findings demonstrate that while the Cu–N_4_ sites are intrinsically responsible for CAT‐ and POD‐like activity, phosphorus modification significantly amplifies both catalytic modes in the CP.

To elucidate the catalytic mechanism underlying the improved CAT and POD performance, first‐principles density functional theory (DFT) calculations were conducted. A schematic representation of the Cu–N_4_ structural modification is shown in Figure 3F, where phosphorus introduced from phytic acid modifications to the Cu center through an etching‐driven process. The optimized reaction pathway and corresponding energy profile for the POD‐like catalytic cycle are shown in Figure 3G,I. Initially, H_2_O_2_ adsorption onto a single‐atom Cu site results in the formation of Cu–H_2_O_2_*, which represents the energy barrier in the pathway (0.76 eV). Subsequent dissociation of H_2_O_2_ produces adsorbed OH*, followed by ·OH desorption. Phosphorus modification lowers the desorption barrier of ·OH, accelerating the conversion of H_2_O_2_ into ·OH and improving the overall POD‐like catalytic efficiency.

For the CAT‐like reaction, four intermediates (*H_2_O_2_, *OH, *O, *O_2_) were identified as adsorbed species on the Cu single‐atom centers (Figure 3H,J). Phosphorus modification increased the affinity for *OH, thus promoting the conversion of H_2_O_2_ to O_2_. The rate‐determining step was calculated to be 0.46 eV. These results indicate that CP preferentially catalyzes H_2_O_2_ dismutation into O_2_ via a Cu–*OH intermediate, confirming its improved CAT‐like catalytic behavior.

### 2.3 Immunomodulation By the CPMR Enhances RT to Trigger ICD and IDO1 Blockade

Only negligible cytotoxicity in MCF‐10A cells was detected following treatment with CPR or CPMR, indicating that both formulations are biocompatible (Figure S5). Because hypoxia within the tumor microenvironment significantly diminishes the therapeutic efficacy of radiotherapy (RT) and given that outstanding CAT‐like activity has been attributed to the CPMR, the potential of the CPMR to relieve hypoxia and thus enhance RT was investigated. As presented in Figure 4A, a significant reduction in HIF‐1α levels under hypoxic conditions was detected upon CPMR treatment according to confocal laser scanning microscopy (CLSM) analysis, indicating that radiosensitization may be facilitated by CPMR via hypoxia alleviation. The IDO1‐inhibitory capacity of CPMR was subsequently examined at the protein level through enzyme‐linked immunosorbent assay (ELISA) and Western blot (WB) analysis (Figure 4B,C). Kyn accumulation was evaluated across the treatment groups, and substantial Kyn suppression was observed, indicating effective inhibition of IDO1 activity. Compared with that in CPR‐treated cells, pronounced downregulation of IDO1 protein expression in 4T1 cells treated with CPMR was detected, demonstrating that significant IDO1 blockade was achieved by CPMR (Figure 4D). These results confirmed that CPMR functions as an efficient IDO1 inhibitor. Furthermore, live/dead costaining of 4T1 cells subjected to different treatments was carried out. As shown in Figure 4E,F, weakened green fluorescence corresponding to live cells and intensified red fluorescence corresponding to dead cells were observed in the CPR+RT and CPMR+RT groups, with the CPMR+RT group exhibiting the greatest level of cell death, which was consistent with the results of the CCK‐8 assay (Figure S6). The synergistic effect of 1‑MT and CPR‑mediated RT after incorporation into CPMR+X‑ray was verified by a combination index of 0.64 (*f_a_* = 0.5) as determined via the Chou‑Talalay method [35], verifying the synergistic effect of 1‐MT and CPR+RT.

**FIGURE 4:**
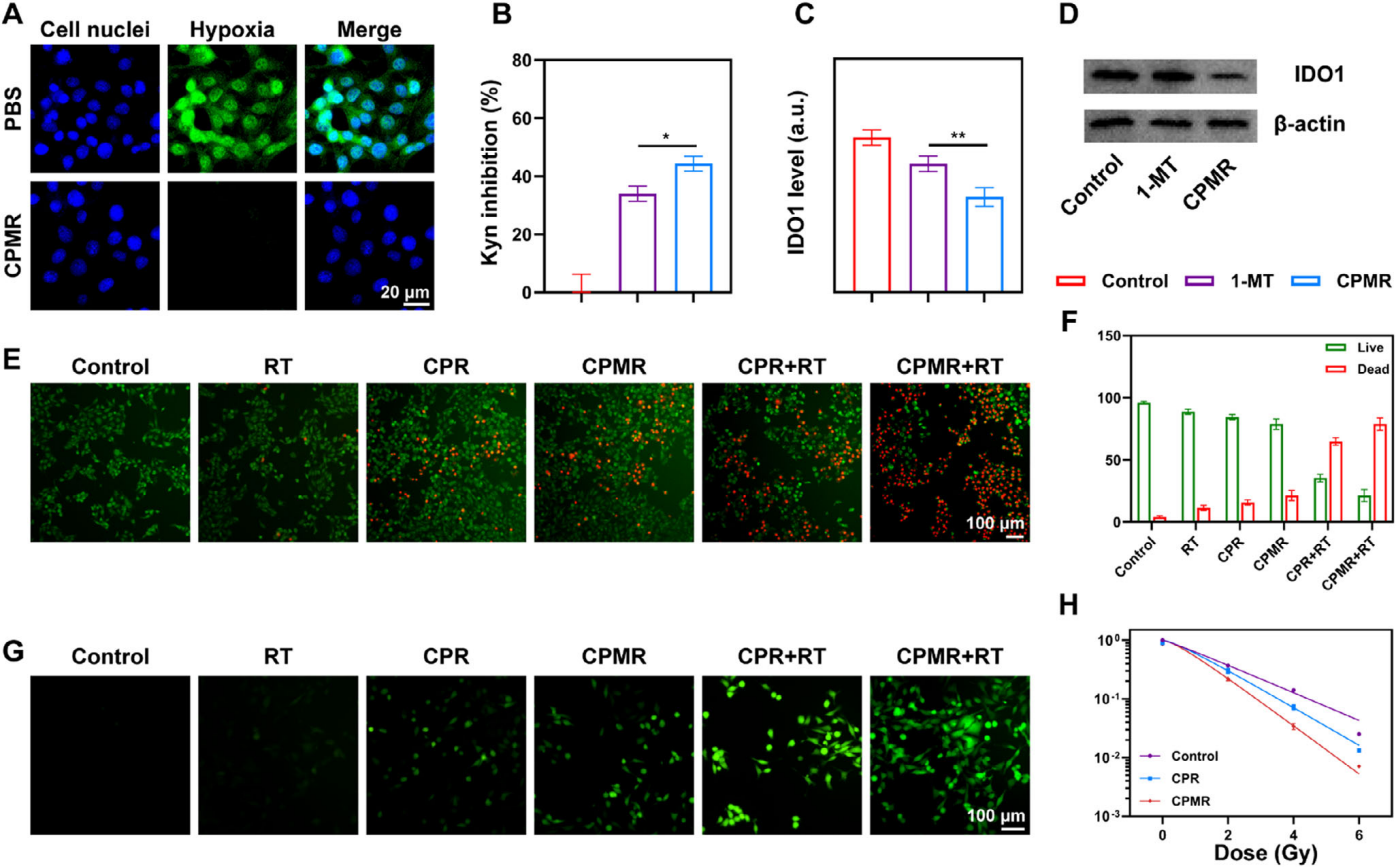
IDO1 blockade and radiosensitization *in vitro*. (A) CLSM images of HIF‐1α staining in 4T1 cells for hypoxic conditions. (B) Kyn inhibition levels were measured via ELISA (n = 3). (C) Quantification analysis and (D) protein level of IDO1 in 4T1 cells measured via WB (n = 3). (E) CLSM images of live/dead cell staining. (F) Quantitative analysis corresponding to the live/dead assay (n = 3). (G) CLSM images of ROS generation using a DCFH‐DA probe. (H) Cell survival fraction fitted via the “multitarget‐single hit” model (n = 3). Statistical significance was determined by one‐way ANOVA with a Tukey post hoc test. ^*^ *P* < 0.05, ^**^ *P* < 0.01.

The intracellular ROS levels were assessed via a DCFH‐DA probe, and the strongest green fluorescence intensity, indicating the highest level of ROS generation, was detected in the CPMR+RT group (Figure 4G). Cell proliferation after treatment was evaluated through colony formation assays. Because clonogenic expansion over multiple generations reflects long‐term cellular viability, colony formation is regarded as the “gold standard” for radiosensitization assessment. As shown in Figure 4H, a dose‐dependent decrease in 4T1 cell survival was observed for both CPR and CPMR. Sensitization enhancement ratios of 1.36 and 1.72 were calculated for CPR and CPMR, respectively. Ionization irradiation triggers DNA double strand breaks, leading to tumorous cells. Thereby, DNA damage was investigated based on γH_2_AX foci formation. As shown in Figure S7, most severe DNA damage was observed in CPMR+RT group compared with other treatment groups. These findings demonstrated pronounced IDO1 blockade and radiosensitization in vitro.

To further elucidate the mechanism by which CPMR improves the efficacy of RT, downstream immune responses were evaluated in vitro. The hallmarks of ICD, namely, precise CRT surface exposure and HMGB1 release, were first examined. As shown in Figure 5A,B, CRT‐associated green fluorescence increased, whereas nuclear HMGB1‐associated red fluorescence decreased in both the RT and CPMR groups, indicating that each monotherapy elicited a moderate ICD response. Quantification of fluorescence intensity further verified these results (Figures S8 and S9). Among all the treatment groups, the CPMR+RT combination induced the most pronounced CRT exposure and HMGB1 release in 4T1 cells. The pretreated 4T1 cells were subsequently cocultured with BMDCs, and DC maturation was analyzed via flow cytometry. The CPMR+RT group presented a significantly elevated proportion of mature DCs and increased production of antitumor cytokines, including tumor necrosis factor‐α (TNF‐α), interferon‐γ (IFN‐γ), and interleukin‐6 (IL‐6) (Figure 5C–G). Moreover, the concentration of extracellular adenosine triphosphate (ATP), another key ICD‐associated signal, was significantly greater in the CPMR+RT group than in the RT alone group (Figure 5H). These results demonstrate that the CPMR augments RT by effectively promoting ICD induction and improving DC maturation, strengthening the antitumor immune response.

**FIGURE 5:**
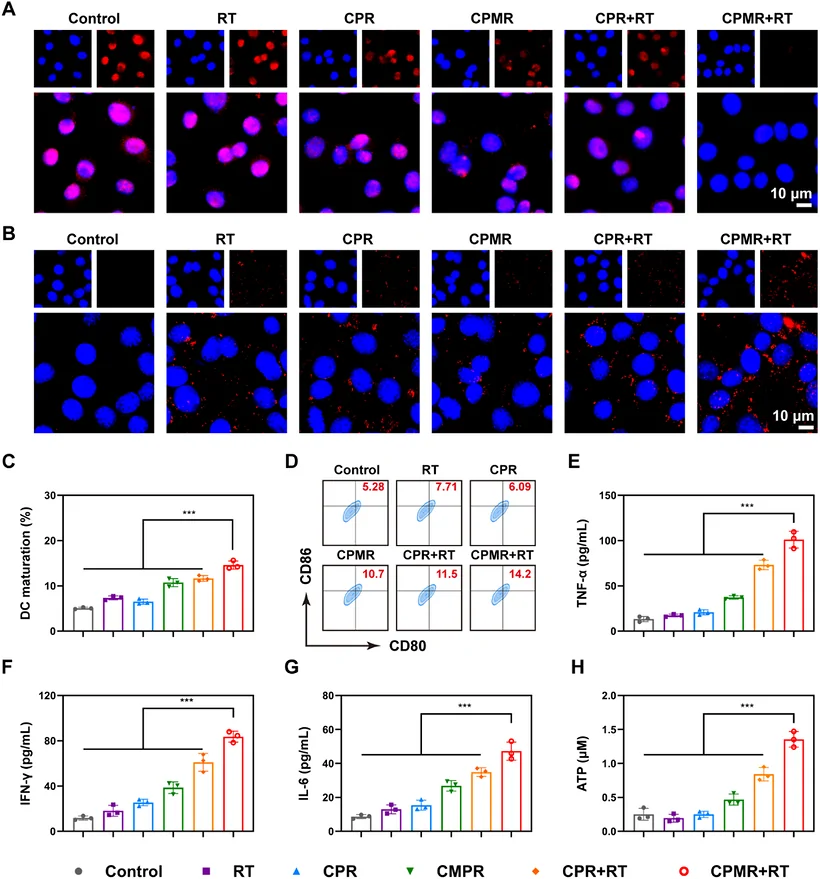
ICD induced by CPMR *in vitro*. (A) CLSM images showing HMGB1 release in 4T1 cells. (B) CLSM images showing CRT exposure in 4T1 cells. (C) Quantification of DC maturation (n = 3). (D) Flow cytometry analysis of mature BMDCs. (E–H) Secretion of proinflammatory cytokines, including (E) TNF‐α, (F) IFN‐γ, and (G) IL‐6 (n = 3). (H) Extracellular ATP production (n = 3). Statistical significance was determined by one‐way ANOVA with a Tukey post hoc test. ^***^ *p* < 0.001.

### 2.4 In Vivo Radiosensitization and Immunosuppression

The systemic toxicity of CPR and CPMR was first evaluated in healthy BALB/C mice. First, pharmacokinetic curve was shown in Figure S10, where it could be included that the majority of both CPR and CPMR was cleared after 24 h. After a month observation period post CPR and CPMR injection, negligible hepatic and renal toxicity was detected (Figure S11). The routine hematological parameters did not significantly differ between the CPR‐ or CPMR‐treated groups and the healthy control group. Histological examination (H&E staining) of major organs further demonstrated the absence of detectable tissue injury or inflammation following administration (Figure S12), indicating favorable systemic biocompatibility.

Given the importance of tumor‐targeting capability in nanozyme‐based antitumor therapy, biodistribution was investigated 24 h after intravenous administration of CY5‐labeled CPM or CPMR in 4T1 tumor‐bearing mice. Ex vivo fluorescence imaging of major organs and tumors revealed that the tumor fluorescence intensity in the CPMR group was 1.98‐fold greater than that in the CPM group, which was attributable to DSPE‐PEG‐RDG camouflage (Figure 6A,B). Moreover, the immunofluorescence intensity of HIF‐1α decreased, as shown in Figure S13, indicating that hypoxia alleviated CPMR. Encouraged by the pronounced tumor‐targeting capacity of the CPMR, an established subcutaneous tumor model was employed to assess antitumor efficacy according to the schedule shown in Figure 6C. The tumors in the control group exhibited rapid, uncontrolled growth. Treatment with RT, CPR, or CPMR alone did not yield substantial tumor inhibition. The CPMR+RT combination resulted in nearly complete tumor eradication, with almost no detectable tumors at the end of the study period (Figure 6D). The body weights remained stable across all the groups (Figure 6E). The final tumor weights confirmed that CPMR+RT had the most pronounced therapeutic effect, resulting in the lowest tumor burden (Figure 6F).

**FIGURE 6:**
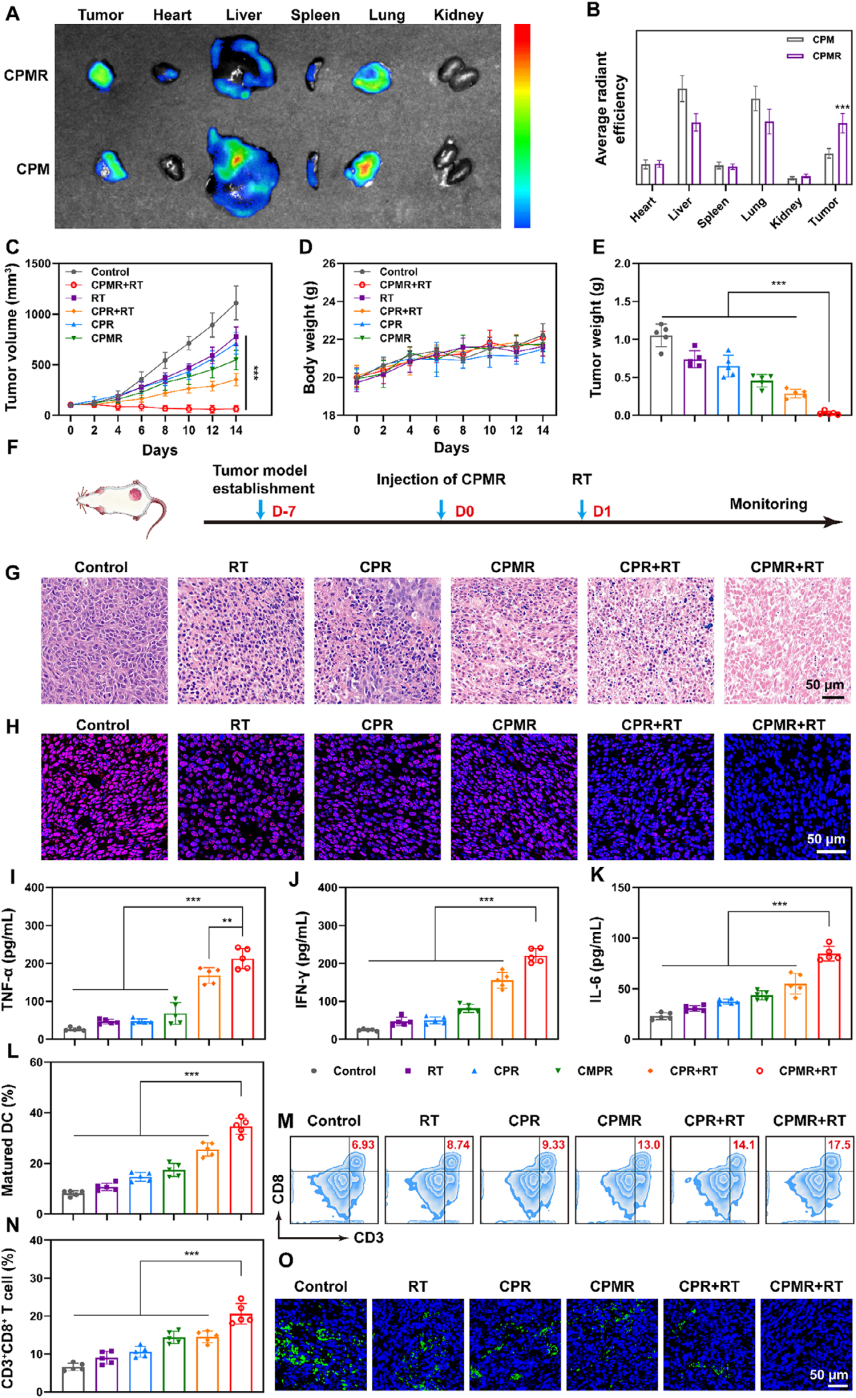
Radiosensitization of the CPMR suppresses tumor growth and triggers a robust immune response. (A) Ex vivo fluorescence images of 4T1 tumor‐bearing mice that received CPM or CPMR. (B) Quantification of fluorescence intensity in the main organs and tumors (n = 5). (C) Schematic of the experimental design. (D) Tumor volume growth curves (n = 5). (E) Body weight monitoring curves (n = 5). (F) Tumor weight (n = 5). (G) HE staining of tumor sections. (H) Immunohistochemical analysis of Ki67. (I) TNF‐α, (J) IFN‐γ, and (K) IL‐6 levels in the serum (n = 5). (L) Quantitative analysis of mature DCs (n = 5). (M) Flow cytometry analysis of CD3^+^CD8^+^ T cells in tumors and (N) quantification analysis (n = 5). (O) Immunofluorescence staining of Treg cells in tumor tissues. Statistical significance was determined by one‐way ANOVA with a Tukey post hoc test. ***P < 0.001.

Histopathological analysis revealed moderate tumor apoptosis in the RT, CPR, and CPMR groups; however, extensive necrosis and apoptosis were observed exclusively in the CPMR+RT group (Figure 6G). Ki67 staining revealed a significant reduction in tumor cell proliferation following CPMR+RT treatment (Figure 6H). Serum proinflammatory cytokines were subsequently quantified. All the treatment groups presented elevated levels of TNF‐α, IFN‐γ, and IL‐6, with the CPMR+RT group showing the most significant increases (Figure 6I–K). Given that these cytokines promote DC maturation and enhance antitumor immunity, intratumoral DC populations were assessed by flow cytometry. As shown in Figure 6L and Figure S14, DC maturation reached 25.9% in the CPR+RT group, whereas the CPMR+RT group exhibited a 2.64‐fold increase compared with RT alone. Intratumoral T‐cell infiltration was subsequently evaluated. Flow cytometry analysis revealed a marked increase in CD8^+^ T‐cell populations in the CPMR+RT group (Figure 6M,N). The proportion of CD4+ T cell in CPMR+RT group was the highest among all groups (Figure S15). Both the CPR+RT and CPMR+RT treatments resulted in reduced Treg frequencies, with CPMR+RT producing a more pronounced inhibitory effect (Figure 6O), underscoring the essential role of 1‐MT in strengthening immune activation.

### 2.5 CPMR‐Enhanced RT for the Treatment of Metastasis and Recurrence

Inspired by the antitumor efficacy and immune response activation observed in previous experiments, an abscopal tumor model was established in BALB/c mice to evaluate whether systemic antitumor immunity could be elicited at distant, nonirradiated tumor sites. Twenty‐four hours after intravenous administration of CPR or CPMR, a 6 Gy irradiation dose was selectively applied to the primary tumor (Figure 7A). As shown in Figure 7B, all the mice in the CPMR+RT group survived until day 40. The growth of both primary and distant tumors was subsequently monitored (Figure 7C,D). In the distant tumor growth curves, rapid tumor progression was observed in the control group, whereas RT, CPR, and CPMR each produced moderate inhibition. Increased tumor suppression was achieved with CPR+RT. The CPMR+RT combination induced the most pronounced inhibition of distant tumor growth, demonstrating a robust abscopal effect.

**FIGURE 7:**
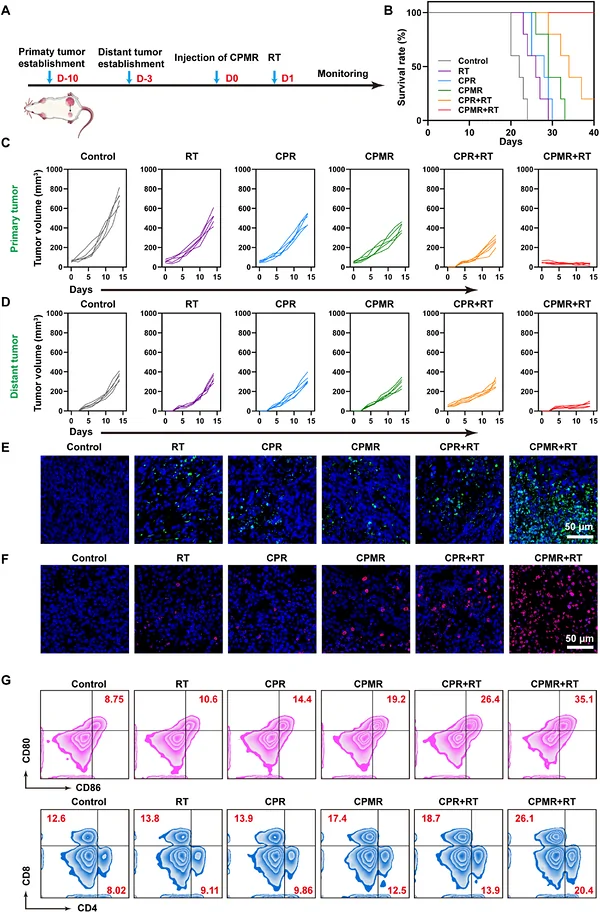
Abscopal effect. (A) Therapeutic schedule for an abscopal model. (B) Survival rates (n = 5). (C) Individual primary tumor growth curves. (D) Individual distant tumor growth curves. (E) TUNEL staining. (F) γ‐H_2_AX staining. (G) Flow cytometry analysis of mature DCs and CD4^+^CD8^+^ T cells in primary tumors.

On day 10 posttreatment, primary and distant tumors were harvested for immunofluorescence staining and flow cytometric evaluation of DC maturation and T‐cell activation. The TUNEL assay revealed greater tumor apoptosis in the CPMR+RT group than in the CPR, CPMR, and RT groups (Figure 7E). Additionally, as shown in Figure 7F, the CPMR+RT group displayed the most intense red nuclear fluorescence in the DSB staining, demonstrating the highest level of DNA damage. As shown in Figure 7G and Figure S16, relative to that in the control group (8.75%), the proportion of CD80^+^CD86^+^ DCs increased to 26.4% in the CPR+RT group and further increased to 35.1% in the CPMR+RT group. Consistent with this finding, CPMR+RT also yielded the highest proportion of CD3^+^CD8^+^ T cells (26.1%) (Figure 7G and Figure S17), representing increases of 2.07‐fold over PBS (12.6%), 1.89‐fold over RT (13.8%), 1.88‐fold over CPMR (13.9%), and 1.40‐fold over CPR+RT (18.7%). Similarly, CPMR+RT produced the greatest expansion of CD4^+^ T cells (Figure S18). These findings indicate that IDO1 inhibition by the CPMR substantially augments RT‐induced immunity, increasing CD8^+^ T‐cell infiltration and cytotoxic T‐lymphocyte activity within tumors, confirming the effective reversal of the immunosuppressive TME through synergistic immune activation.

Given that central memory T (TCM) cells act as critical reservoirs for sustaining long‐term immune protection, their involvement was assessed via a tumor‐recurrence model following the protocol outlined in Figure 8A. Five days after surgical removal of the tumors, tumor recurrence was monitored (Figure 8B). In addition, greater Kyn inhibition efficacy was observed in the CPMR+RT group (Figure 8C). Flow cytometric analysis revealed significantly elevated proportions of TCMs within both the CD4^+^ and CD8^+^ T‐cell populations compared with those in the controls (Figure 8D,E). No detectable tumor regrowth was observed in the CPMR+RT group, while under CPMR+RT treatment, the mice survived longer than those in the control group did (Figure 8F). Immunofluorescence staining of CD8^+^ T cells also verified the increase in CD8^+^ T cells in tumors from the CPMR+RT group, as shown in Figure 8G. These results indicate that CPMR‐enhanced RT not only suppresses primary and distant tumor growth but also establishes durable immunological memory capable of preventing recurrence.

**FIGURE 8:**
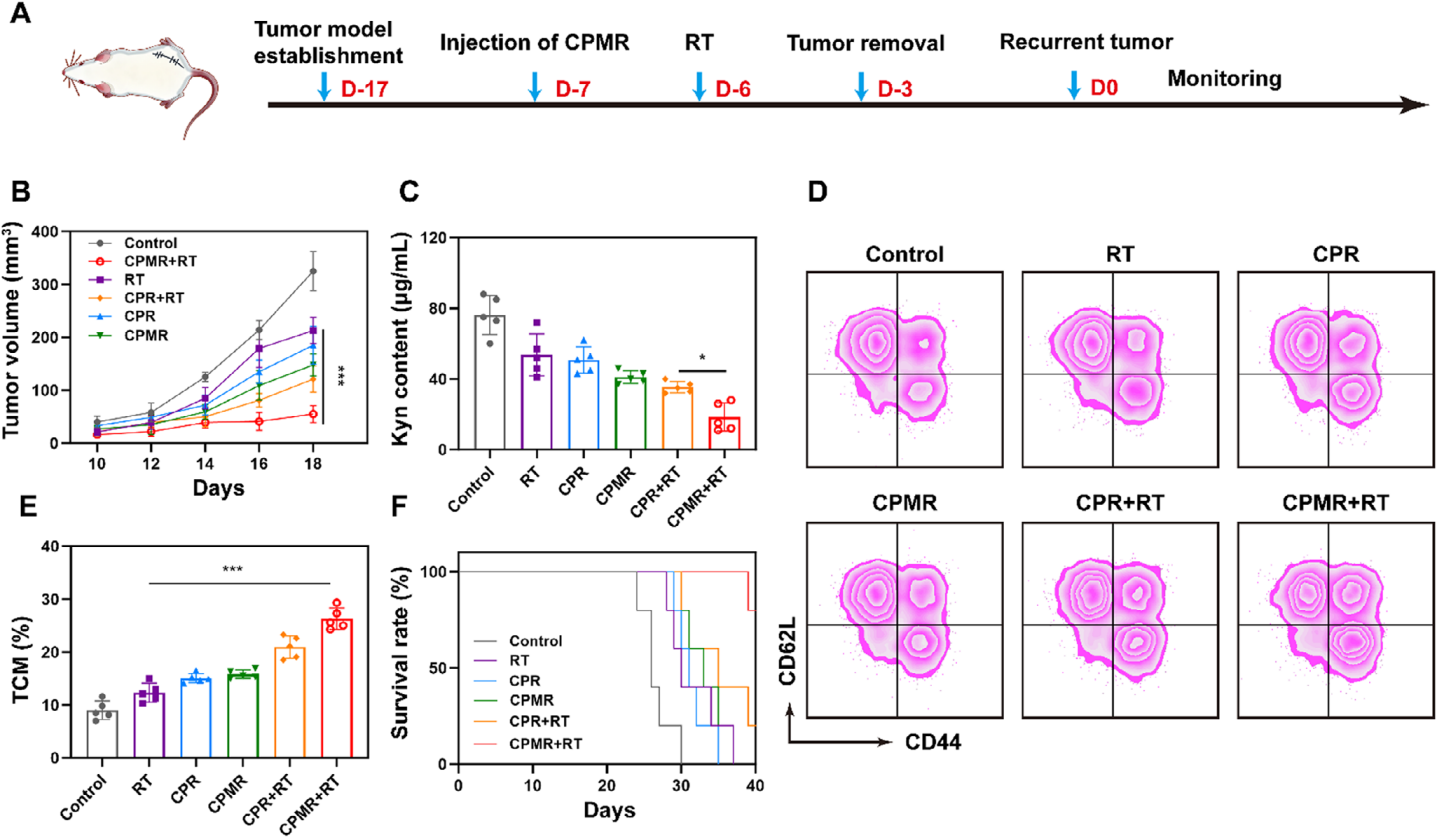
Recurrence inhibition. (A) Therapeutic schedule for a recurrence model. (B) Tumor volume (n = 5). (C) Kyn inhibition in tumors (n = 5). (D) Flow cytometry analysis of TCM cells in recurrent tumors and (E) quantification analysis (n = 5). (F) Survival rate (n = 5). Statistical significance was determined by one‐way ANOVA with a Tukey post hoc test. ^*^ *P* < 0.05, ^***^ *P* < 0.001 .

To benchmark our strategy against the current clinical standard, we included an aPD‑1+RT group and compared it with CPMR+RT in the same tumor model (Figure S19A). Tumor growth curves showed that CPMR+RT significantly reduced tumor volume compared with either RT alone or aPD‑1+RT (Figure S19B). Analysis of immune responses revealed that CPMR+RT yielded a higher frequency of mature dendritic cells (Figure S19C,E) and greater CD8^+^ T cell infiltration (Figure S19D,F) than the aPD‑1+RT regimen. Consistently, Ki67 staining indicated the lowest proliferative activity in the CPMR+RT group (Figure S19G). Taken together, these data demonstrate that CPMR combined with radiotherapy outperforms the standard aPD‑1 plus radiotherapy combination in both antitumor immunity and tumor growth control.

## 3 Discussion

In this work an “all‐in‐one” single atom Cu nanozyme termed CPMR was developed for synergistic radiotherapy in breast cancer. Owing to the P‐doped single‐atom Cu–N_4_ catalytic sites, CPMR exhibits dual enzyme‐mimicking activities of CAT and POD, enabling ROS generation and hypoxia alleviation in the TME. Meanwhile, CPMR was loaded with IDO1 inhibitor 1‐MT and endowed with tumor specific targeting ability by RGD modification. The CP nanozyme acts as the core component for generating reactive oxygen species and alleviating hypoxia. Incorporation of 1‐MT enhances immune responses, while RGD modification improves tumor accumulation. Mechanistically, CPMR combined with radiotherapy enhances tumor killing by inducing immunogenic cell death, alleviating hypoxia, and synergistically activating dendritic cells and CD8^+^ T cells via IDO1 inhibition and downregulation of regulatory T cells.

Although in this study we introduced a novel single atom nanozyme for radiosensitization and immune response activation, several challenges remain for clinical translation. The 4T1 tumor bearing model on mice does not fully reflect tumor heterogeneity. Redundant immune evasion pathways limit IDO1 blockade alone, and the benefit of our triple‑mechanism platform over current standards such as radiotherapy plus anti‑PD‑1/PD‑L1 treatment requires direct comparison. Synthesis of the P‑modified single‑atom Cu nanozyme is complex. Meanwhile, large‑scale production, batch consistency, long‑term stability and sterilization remain technical bottlenecks. Longer term toxicity still need further evaluation. Given the limitations of the 4T1 model, future studies using humanized PDX models are needed to further evaluate the translational potential of CPMR to enhance radiosensitivity. In the future research, we will focus on synthesis optimization, quality control, extended safety observation and combination with immune checkpoint inhibitors of CPMR.

## 4 Conclusion

To improve the catalytic performance of SAzymes, a Cu‐based SAzyme incorporating phosphorus modification, designated CPMR, was developed in this study. The dual enzymatic functions of this Cu‐SAzyme act synergistically: its catalase‐mimicking activity generates oxygen to mitigate tumor hypoxia and augment radiosensitization, whereas its peroxidase‐mimicking activity induces substantial oxidative stress, triggering robust ICD. The ensuing ICD activated both the innate and adaptive immune pathways, promoting strong recruitment and activation of tumor‐infiltrating cytotoxic T lymphocytes at the primary tumor site. The controlled release of the IDO1 inhibitor 1‐MT from the nanozyme effectively suppressed the kynurenine pathway, reversing aberrant tryptophan catabolism and limiting the accumulation and immunosuppressive function of regulatory T cells. The resulting immune activation was both systemic and durable. The combined treatment induced a pronounced abscopal effect, suppressed the growth of distant, nonirradiated tumors, and established a protective immunological memory capable of preventing tumor recurrence. These therapeutic outcomes are driven by the successful reprogramming of the immunosuppressive TME. Through the concurrent enhancement of direct tumor cytotoxicity via radiosensitization, the induction of systemic antitumor immunity through ICD, and the disruption of critical immunosuppressive mechanisms, the CPMR nanozyme effectively transformed the inherent dual immunomodulatory effects of radiotherapy into a potent and sustained antitumor response. These results position the CPMR as a promising platform for integrated and effective radioimmunotherapy against breast cancer while further investigations are warranted to address key translational challenges such as evaluation of therapeutic efficacy on PDX models.

## Conflicts of Interest

The authors declare no conflicts of interest.

## Supporting information




**Supporting File**: advs78239‐sup‐0001‐SuppMat.docx

## Data Availability

The data that support the findings of this study are available from the corresponding author upon reasonable request.
