# Supplementary material for: An Activity‐Reinforced P‐Modified Single‐Atom Cu Nanozyme Potentiates IDO Blockade‐Induced Tumor Radiotherapy
Source: Adv Sci (Weinh). 2026 Oct 31:e24253. Online ahead of print. doi: 10.1002/advs.202524253 (PMC13723725; doi:10.1002/advs.202524253)
Supplement: Supplementary file 1 [file ADVS-9999-e24253-s001.docx]

**Supporting information**

**An activity-reinforced P-modified single-atom Cu nanozyme potentiates IDO blockade-induced tumor radiotherapy**

Wei Zhao^1^, Songliu Hu^2^, Zhanggui Wang^3^, Liqun Wang^1^, Liming Zhang^1^, Qi Li^2^, Hua Tang^2^, Yang Zhong^5^, Zhongxian Zhang^4^, Kelong Fan^6,*^, Shipeng Ning^2,*^, Xiyun Yan^6,*^

^1^Research Center of Nanomedicine Technology, The Second Affiliated Hospital of Guangxi Medical University, Nanning, 530000, China.

^2^Harbin Medical University Cancer Hospital, Harbin, 150000, China.

^3^Department of Radiotherapy, Cancer Center, Aerospace Center Hospital, Beijing 100049, China.

^4^State Key Laboratory of Metabolic Dysregulation & Prevention and Treatment of Esophageal Cancer, Tianjian Laboratory of Advanced Biomedical Sciences, Academy of Medical Sciences, Zhengzhou University, Zhengzhou 450052, China.

^5^Department of Radiation Oncology, Anhui No. 2 Provincial People's Hospital, Hefei, 230031, China.

^6^CAS Engineering Laboratory for Nanozyme, State Key Laboratory of Biomacromolecules Institute of Biophysics, Chinese Academy of Sciences, Beijing 100101, China.

E-mail: [fankelong@ibp.ac.cn](mailto:fankelong@ibp.ac.cn); [nspdoctor@sr.gxmu.edu.cn](mailto:nspdoctor@sr.gxmu.edu.cn); [yanxy@ibp.ac.cn](mailto:yanxy@ibp.ac.cn).

**Experimental Section**

1. **Chemicals**

ZnNO_3_·6H_2_O (97%), 2-methylimidazole (2-MI), and copper acetylacetonate (97%) were purchased from Sigma‒Aldrich. Phytic acid (98%) was obtained from Energy Chemical. Hexadecyl trimethyl ammonium bromide (CTAB, 99%) and 1-methyl-DL-tryptophan (1-MT, 97%) were obtained from Aladdin-Rich. Ethyl silicate (TEOS, AR) was purchased from Sinopharm.

2. **Fabrication of the CPR, CPM and CPMR**

ZnNO_3_·2H_2_O was dissolved in methanol, while 2-MI and copper acetylacetonate were dissolved in methanol. The two solutions were then mixed and stirred vigorously for 24 hours. The resulting product was centrifuged and washed with methanol. The obtained product was vacuum-activated at 80 °C for 24 hours, subsequently dissolved in water, and mixed with phytic acid under stirring for half an hour. After centrifugation and washing, the product was obtained. The product was ultrasonically dispersed in methanol, and an appropriate amount of CTAB solution (25 mg/ml), NaOH solution (6 mg/ml), or TEOS (1.2 ml) was added. The resulting dispersion was stirred for 2 hours. The product was then centrifuged and washed with ethanol, followed by vacuum drying to obtain CP.

1-MT was dissolved in water, and an appropriate amount of CP suspension was added. The mixture was stirred overnight to obtain CPM. DSPE-PEG-RGD was dissolved in water, and an appropriate amount of CPM was added. After ultrasonication, the product was centrifuged and washed with ethanol to obtain CPMR.

The CP solution was mixed with the DSPE-PEG-RGD solution. After ultrasonication, the product was centrifuged and washed with ethanol to obtain the CPR.

3. **Characterization**

The morphology of the CPMR samples was characterized via TEM/HAADF-STEM (JEOL JEM-2100F, 200 kV). The zeta potentials were measured via DLS (Malvern Zetasizer Nano ZS) in ddH_2_O. XPS spectra (Thermo Scientific ESCALAB 250Xi, Al‒Kα source) calibrated to C 1 s (284.8 eV) were obtained for surface elemental analysis. The UV‒vis absorption spectra were recorded on a UH4150 (HITACHI) spectrophotometer. The Fourier transform infrared (FTIR) spectrum was recorded on an FTIR spectrometer (Spectrum One, Perkin Elmer) via the standard KBr pellet technique.

4. **POD and CAT-like activity assessment**

POD-like: 10 µL of TMB ethanol solution (10 mg/mL) was added to 990 µL of CMR or CPMR solution containing 10 mM H_2_O_2_ (10 µL). After 5 min, the catalytic oxidation of TMB was studied by UV‒Vis absorption spectroscopy. Kinetic assays of CMR and CPMR with H2O2 as the substrate were performed by adding 1 mM TMB and different amounts of H_2_O_2_ solution. The oxidation of TMB was measured through recording the absorption variations at 652 nm. The Michaelis‒Burk constant was calculated via the Lineweaver‒Burk plot: 1/V=km/Vmax (1/[S]+1/km).

CAT-like: CMR and CPMR were dispersed in H_2_O_2_ solution. Next, the oxygen levels in the solutions were measured with a portable dissolved oxygen meter (JPSJ-606 L, Leici).

5. **Cell viability evaluation**

In vitro cytotoxicity was evaluated via a cell counting kit-8 (CCK-8) assay. 4T1 cells were plated in a 96-well plate at a density of 10^4^ cells per well and cultured until 80% confluence. Then, the medium was replaced with various concentrations of CPR or CPMR for another 24 h, after which the cells were washed with PBS, the medium was discarded, and fresh 10% CCK-8 medium was added and cultured for another 1 h. Finally, the 450 nm absorbance was recorded on a microplate reader (Spark 10 M, Tecan, Switzerland).

The viability of the cells in different treatment groups was detected. After 24 h of culture, the medium was replaced with fresh medium containing different amounts of CPR or CPMR. Then, the cells in the RT treatment group were irradiated with 6 Gy. After 24 h of incubation, the cells were washed once with PBS and further incubated with 100 μL of CCK-8 for 1 h. The absorbance at 450 nm was then measured with a microplate reader.

Chou-Talalay’s isobolographic method was calculated for CPR-based RT and 1-MT on CPMR+X-ray using the equation combination index (CI) using previously reported method [1]. The effect fraction (*f_a_*) is the cell death measured by the CCK8 method. Using the more conservative reciprocal assumption (α = 0), CI < 1, CI = 1 and CI > 1 indicate synergistic, additive and antagonistic effects, respectively.

6. **Western blot analysis of the IDO1 protein**

After the protein concentration was determined via the bicinchoninic acid method, WB analysis was carried out with an anti-IDO1 mouse antibody (Proteintech). Protein band intensity analysis was performed via ImageJ software to quantify the level of each protein by determining the grayscale level of each band and normalizing it to that of β-actin.

7. **Evaluation of** **Kyn inhibition via ELISA**

After the 4T1 cells were cultured under different conditions for 24 h, the culture medium was removed, and 10 mL of serum-free DMEM was added. A KYN ELISA kit (YJ243862; Enzyme-Linked Biotechnology, China) was used to analyze the supernatant samples following the manufacturer's instructions.

8. **Intracellular reactive oxygen species production**

The DCFH-DA probe was used to test the ROS level. First, 4T1 cells were cultured in a culture dish (5 × 10^4^ cells per well) for 24 h. Then, the cells were subjected to various treatments, including control, RT (6 Gy), CPR (50 μg/mL), CPMR (50 μg/mL), CPR (50 μg/mL) + RT (6 Gy), and CPMR (50 μg/mL) + RT (6 Gy). The cells were washed with PBS and incubated with 1 μL of DCFH-DA (10 mM) at 37 °C for 20 min. Afterward, fluorescence imaging was recorded via confocal laser scanning microscopy (CLSM).

9. **Live/dead assay**

Calcein AM and PI staining was performed to detect cell death. 4T1 cells were seeded in 6-well plates at a density of 1×10^5^ cells per well and cultured for 24 h. The cell medium was then replaced with fresh medium containing 50 μg/mL CPR or CPMR. After exposure for 4 h, the cells were irradiated with an 808 nm laser for 5 min and further incubated for 16 h. The cells were washed with PBS and stained with PI and calcein-AM. Live/dead imaging was monitored via a fluorescence microscope.

10. **Detection of HIF-1α expression in vitro**

4T1 cells were seeded in 6-well plates at a density of 1×10^5^ cells per well and cultured overnight. Then, the cells were treated with PBS, CPR or CPMR under hypoxic conditions. After incubation for 24 h under hypoxia, the cells were harvested and stained with a fluorescently labeled HIF-1α antibody. Then, CLSM was used for monitoring.

11. **Immunogenic cell death markers and DC maturation assay**

To evaluate immunogenic cell death (ICD), 4T1 cells were seeded in 6-well plates and subjected to various treatments. To detect immunosuppressive cell death markers, CRT/HMGB1 immunofluorescence staining was performed by fixing cells with 4% PFA, permeabilizing them with 0.1% Triton X-100, and then staining them with anti-CRT (Abcam ab2907, 1:200) or anti-HMGB1 (Abcam ab79823, 1:500) antibodies overnight at 4 °C, followed by incubation with Alexa Fluor-conjugated secondary antibodies. Image fluorescence was observed via confocal microscopy.

12. **In vitro DC maturation assay**

Bone marrow-derived dendritic cells (BMDCs) were isolated from 6-week-old male BALB/c mice via established protocols. After adherence, immature DCs were harvested for subsequent experiments. To assess the immunological effects, a Transwell coculture system was established. 4T1 cells were seeded in the upper chambers, while DCs were seeded in the lower chambers. Following 4 h of culture for adhesion, the 4T1 cells received the designated treatments. The treated tumor cells were then cocultured with DCs to evaluate immune activation. Following 24 h of coculture, the DCs were harvested and stained with anti-mouse CD11c-APC, anti-CD80-PE, and anti-CD86-PE-Cy7 antibodies. Flow cytometry analysis was performed by gating on CD11c+ populations. For TNF-α/IFN-γ/IL-6/ATP release analysis, cell supernatants were collected and evaluated via ELISA kits (Beyotime). The controls included unstained cells for background subtraction.

13. **Antitumor efficacy in a subcutaneous model**

BALB/c mice were purchased from Beijing Vital River Company. All animal operations were performed in accordance with the guidelines and regulations of the Ethics Committee of the Second Affiliated Hospital of Guangxi Medical University.

To establish tumors, 100 µL of a 4T1 cell suspension was injected subcutaneously into the right lap. When the tumor volume reached 100 mm^3^, 6-week-old female BALB/C mice inoculated with 4T1 cells were randomly divided into 6 groups with 5 mice in each group: control, RT (6 Gy), CPR (50 μg/mL), CPMR (50 μg/mL), CPR (50 μg/mL) + RT (6 Gy), and CPMR (50 μg/mL) + RT (6 Gy), and the tumor volume was measured every two days via digital calipers. The tumor volume (V) was calculated as follows: V = (length × width2)/2. At the experimental endpoint, the tumors were resected, photographed, and weighed. The tissue samples were immediately fixed in 4% paraformaldehyde for 24 h and then processed through a graded ethanol series and xylene before being embedded in paraffin. Sections (4 µm thick) were mounted on charged slides for sequential histological analysis and immunofluorescence staining. Next, for immune response detection, fresh tumor tissues were isolated into single-cell suspensions with the following fluorescent antibodies: anti-CD3-APC and anti-CD8-PE for T cells and anti-CD11b-APC, anti-CD80-PE and anti-CD86-PE-Cy7 antibodies for mature DCs. These cells were then analyzed by flow cytometry according to the abovementioned methods. The contents of ATP and cytokines (including IL-10, TGF-β1, TNF-α, and IFN-γ) in the serum were determined via ELISA kits according to the manufacturer's instructions.

14. **Antitumor efficacy in a bilateral tumor model**

To construct a bilateral tumor model, female BALB/c mice received subcutaneous injections of 4T1 cells in the right lap to form primary tumors and 4T1 cells in the left lap to form abscopal tumors. When the primary tumors reached 100 mm3, the mice were divided into the following treatment groups: control, RT (6 Gy), CPR (50 μg/mL), CPMR (50 μg/mL), CPR (50 μg/mL) + RT (6 Gy), and CPMR (50 μg/mL) + RT (6 Gy). Irradiation was applied 24 h after the injection. At the end of observation, the excised tumors were embedded in paraffin for immunofluorescence analysis. Single-cell suspensions from tumors were stained with the following fluorescent antibodies: anti-CD4-APC and anti-CD8-PE for T cells and anti-CD11b-APC, anti-CD80-PE and anti-CD86-PE-Cy7 antibodies for DCs. Then, flow cytometry analysis was conducted to assess the proportions.

15. **Antitumor efficacy in a recurrence model**

A recurrence model was established via the injection of 4T1 tumor cells into the right laps of BALB/c mice, followed by various treatments. Three days after the tumors were excised by surgery, 4T1 cells were injected into the excised part to simulate tumor recurrence. The recurrent tumors from the mice in the 2 groups were then collected to isolate single cells for flow cytometry analysis of T_CM_.

Tumor tissues were harvested from euthanized mice and washed with pre-cooled phosphate buffered saline (PBS). After removing necrotic tissues and surrounding connective tissues, tumor samples were minced and digested with tissue dissociation buffer under gentle shaking. Single-cell suspensions were obtained via mesh filtration and centrifugation. Red blood cells were lysed using lysis buffer, and the remaining cells were resuspended and counted. For surface marker staining, cells were incubated with fluorescent antibodies against CD4 and CD25 in the dark on ice. Subsequently, cells were fixed and permeabilized using a commercial transcription factor staining buffer set. Intracellular staining of Foxp3 was then performed under dark conditions. Finally, stained cells were resuspended and detected by flow cytometry. Treg cells were identified as CD4^+^CD25^+^Foxp3^+^ subsets and their proportions were analyzed.

16. **Pathological evaluations**

The in vivo biosafety assessment was performed via routine blood and biochemical tests on the mice. After 1 month of treatment, three groups of mice were randomly selected for blood analysis. For histological analysis, the mice were sacrificed after treatment, and the organs in each group were separated. H&E staining was performed by slicing the tissue. Major organs were dissected to prepare paraffin sections for H&E staining to assess systemic toxicity.

17. **Statistics**

The data are expressed as the means ± SD. Statistical significance was determined by one-way ANOVA with a Tukey post hoc test. The data were classified with p values and denoted by (*) for p < 0.05, (**) for p < 0.01, and (***) for p < 0.001.

Figure S1. TEM image of CP.

Figure S2. TEM image of CPR.

Figure S3. FTIR transmittance spectra of CPMR after one week of incubation in simulated tumor microenvironment.

Figure S4. 1-MT releasing behavior profile of CPMR (n=3).

**Figure S5.** Cell viability of MCF-10A cells in the presence of various concentrations of CPR or CPMR (n=3).

**Figure S6.** Viability of 4T1 cells in different groups (n=3).

**Figure S7.** γ-H_2_AX foci formation correlates with DNA damage.

**Figure S8.** Relative HMGB1 fluorescence intensity (n=3).

**Figure S9.** Relative CRT fluorescence intensity (n=3).

**Figure S10.** Pharmacokinetic parameters of CPR and CPMR respectively (n=5).

**Figure S11.** Hematology parameters. (A) White blood cell (WBC), (B) platelet (PLT), (C) red blood cell (RBC), (D) hemoglobin (HGB), (E) mean corpuscular volume (MCV), (F) mean corpuscular hemoglobin (MCH) and (G) mean corpuscular hemoglobin concentration (MCHC) (n=5).

**Figure S12.** Representative HE staining of main organs.

**Figure S13.** Immunofluorescence staining of HIF-1α.

**Figure S14.** DC maturation in tumors.

**Figure S15.** Flow cytometry analysis of CD3^+^CD8^+^ T cells in tumors.

**Figure S16.** Proportion of matured DC (n=5). Statistical significance was determined by one-way ANOVA with a Tukey post hoc test. **p < 0.01, ***p < 0.001, ****p < 0.0001.

**Figure S17.** Proportion of CD8^+^ T cells (n=5). Statistical significance was determined by one-way ANOVA with a Tukey post hoc test. **p < 0.01, ***p < 0.001, ****p < 0.0001.

**Figure S18.** Proportion of CD4^+^ T cells (n=5). Statistical significance was determined by one-way ANOVA with a Tukey post hoc test. **p < 0.01, ***p < 0.001, ****p < 0.0001.

**Figure S19.** Antitumor effect compared with aPD-1+RT strategy. (A) Schematic procedure of treatment. (B) Tumor volume variation during observation (n=5). (C) Matured DC proportion in each group (n=5). (D) Proportion of CD3^+^CD8^+^ T cells (n=5). (E) Flow cytometry analysis of matured DC. (F) Flow cytometry analysis of CD3^+^CD8^+^ T cells. (G) Immunofluorescence staining of Ki67 in tumor tissues. Statistical significance was determined by one-way ANOVA with a Tukey post hoc test. **p < 0.01, ***p < 0.001, ****p < 0.0001.

**References**

[1]	Y. He, H. Chen, W. Li, L. Xu, H. Yao, Y. Cao, Z. Wang, L. Zhang, D. Wang, D. Zhou, *Journal of Nanobiotechnology* **2023**, *21* (1), 209, <https://doi.org/10.1186/s12951-023-01970-8>.
